# Supplementary material for: A blueprint for robust crosslinking of mobile species in biogels with weakly adhesive molecular anchors
Source: Nat Commun. 2017 Oct 10;8:833. doi: 10.1038/s41467-017-00739-6 (PMC5635012; doi:10.1038/s41467-017-00739-6)
Supplement: Supplementary file 1 — Supplementary Information [file 41467_2017_739_MOESM1_ESM.pdf]

Supplementary Figures

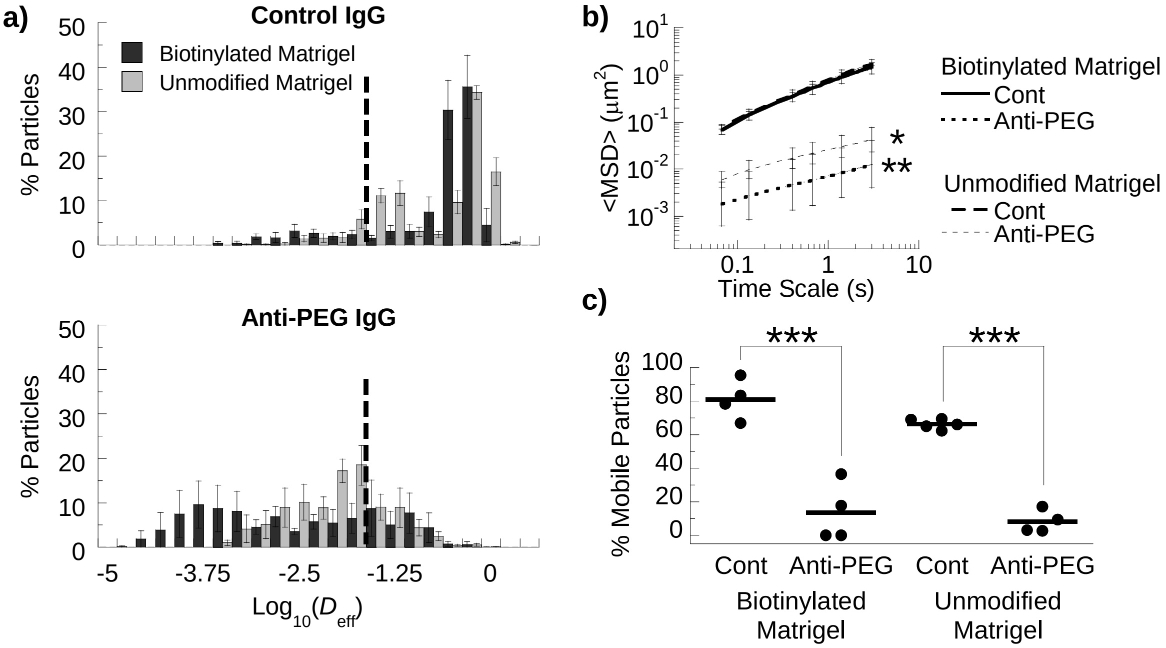

Supplementary Figure 1: Dependence of matrix biotinylation on trapping
Diffusion of PEG-conjugated nanoparticulates in unmodified or biotinylated Matrigel® treated with anti-PEG IgG or control IgG. **(a)** Distributions of the mean logarithms of individual particle effective diffusivities (D_{eff}) at a time scale of 0.2667 s. Log (D_{eff}) values to the left of the dashed line correspond to particles with displacements of less than 100 nm (i.e., roughly the particle diameter) within 0.2667 s. **(b)** Ensemble-averaged geometric mean square displacements (<MSD>) as a function of time scale. **(c)** Fraction of mobile nanoparticulates in Matrigel® treated with different IgG. N=4-5 separately prepared slides/condition with 83-528 particles tracked per slide. Error bars represent standard error of the mean (SEM). *p<0.05 **p<0.01 ***p<0.001 as calculated by repeated measures two-way ANOVA compared to control.

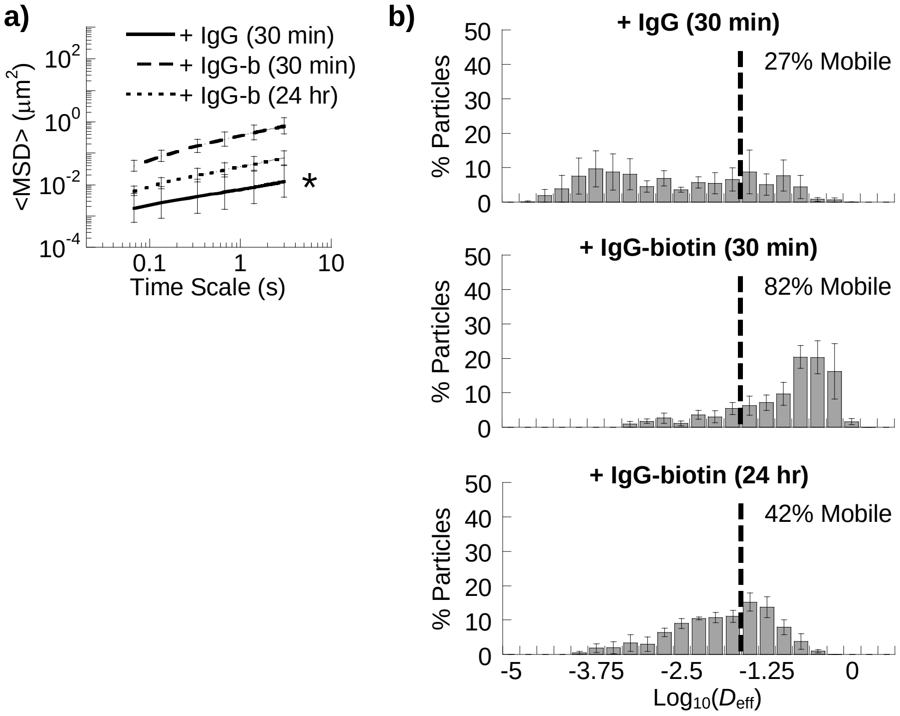

Supplementary Figure 2: Dependence of trapping ability on incubation time
Diffusion of PEG-conjugated nanoparticulates in biotinylated Matrigel® modified with neutravidin, treated with either unmodified anti-PEG IgG or biotinylated anti-PEG IgG incubated for 30 min or 24 hr was measured. **(a)** Ensemble-averaged geometric mean square displacements (<MSD>) as a function of time scale. **(b)** Distributions of the mean logarithms of individual particle effective diffusivities (D_{eff}) at a time scale of 0.2667 s. Log (D_{eff}) values to the left of the dashed line correspond to particles with displacements of less than 100 nm (i.e., roughly the particle diameter) within 0.2667 s. N=3-4 separately prepared slides/condition with 61-174 particles tracked per slide. Error bars represent standard error of the mean (SEM). *$p<0.05$ as calculated by repeated measures two-way ANOVA compared to control.

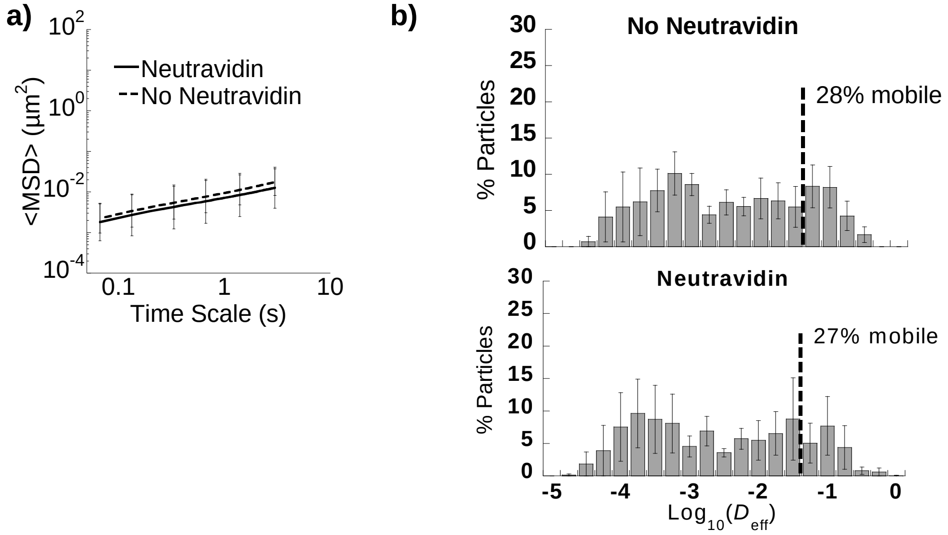

Supplementary Figure 3: Dependence of trapping ability of IgG on neutravidin
Diffusion of PEG-conjugated nanoparticulates in biotinylated Matrigel® treated with anti-PEG IgG, with or without neutravidin was measured. **(a)** Ensemble-averaged geometric mean square displacements (<MSD>) as a function of time scale. **(b)** Distributions of the mean logarithms of individual particle effective diffusivities (D_{eff}) at a time scale of 0.2667 s. Log (D_{eff}) values to the left of the dashed line correspond to particles with displacements of less than 100 nm (i.e., roughly the particle diameter) within 0.2667 s. N=4 separately prepared slides/condition with 92-174 particles tracked per slide. Error bars represent standard error of the mean (SEM). P-value was calculated by repeated measures two-way ANOVA.

Supplementary Tables

Antibody	$K_{D,1}$ (M)	$K_{D,2}$ (M)
IgG	4.0×10^{-8}	
IgG-biotin	9.4×10^{-9}	3.3×10^{-12}

Supplementary Table 1. Dissociation constants of (i) native anti-PEG IgG and (ii) biotinylated anti-PEG IgG to biotinylated Matrigel® treated with neutravidin, measured using BLI.

Antibody	K_D (M)	k_{on} (M^{-1} s^{-1})	k_{off} (s^{-1})
IgG	9.6×10^{-9}	8.9×10^{3}	8.5×10^{-5}
IgG-biotin	4.3×10^{-9}	2.1×10^{4}	8.9×10^{-5}
IgG-biotin + neutravidin	8.1×10^{-10}	7.0×10^{4}	5.7×10^{-5}

Supplementary Table 2. Binding kinetics and dissociation constants of (i) native anti-PEG IgG, (ii) biotinylated anti-PEG IgG, and (iii) biotinylated anti-PEG IgG complexed with neutravidin, to PEG, measured using BLI.
