# Supplementary material for: A blueprint for robust crosslinking of mobile species in biogels with weakly adhesive molecular anchors
Source: Nat Commun. 2017 Oct 10;8:833. doi: 10.1038/s41467-017-00739-6 (PMC5635012; doi:10.1038/s41467-017-00739-6)
Supplement: Supplementary file 3 — Description of Additional Supplementary Files [file 41467_2017_739_MOESM3_ESM.pdf]

Description of Additional Supplementary Files

File Name: Supplementary Movie 1
Description: 100 nm PEG-conjugated nanoparticulates in biotinylated Matrigel®

File Name: Supplementary Movie 2
Description: 100 nm PEG-conjugated nanoparticulates in biotinylated Matrigel® with antiPEG IgG

File Name: Supplementary Movie 3
Description: Respiratory Syncytial Virus in Matrigel®

File Name: Supplementary Movie 4
Description: Respiratory Syncytial Virus in Matrigel® with Synagis, a cocktail of three antiRSV antibodies
